# Effects of Stocking Density on the Growth Performance, Physiological Parameters, Antioxidant Status and Lipid Metabolism of *Pelteobagrus fulvidraco* in the Integrated Rice-Fish Farming System

**DOI:** 10.3390/ani13111721

**Published:** 2023-05-23

**Authors:** Weixu Diao, Rui Jia, Yiran Hou, Yin Dong, Bing Li, Jian Zhu

**Affiliations:** 1 Wuxi Fisheries College, Nanjing Agricultural University, Wuxi 214081, China; 2021113006@stu.njau.edu.cn (W.D.); jiar@ffrc.cn (R.J.); houyr@ffrc.cn (Y.H.);; 2 Key Laboratory of Integrated Rice-Fish Farming Ecology, Ministry of Agriculture and Rural Affairs, Freshwater Fisheries Research Center, Chinese Academy of Fishery Sciences, Wuxi 214081, China; 3 International Joint Research Laboratory for Fish Immunopharmacology, Freshwater Fisheries Research Center, Chinese Academy of Fishery Sciences, Wuxi 214081, China

**Keywords:** physiological stress response, oxidative stress, metabolism function, immune response

## Abstract

**Simple Summary:**

*Pelteobagrus fulvidraco* is a common freshwater fish, mainly distributed in China, Vietnam, Laos, and southeast Siberia of Russia. Its meat is delicious and has high nutritional value. The purpose of the study was to explore the suitable farming density of *P. fulvidraco* in integrated rice–fish farming. Our study revealed that the stocking density of *P. fulvidraco* in a paddy field should not exceed 250 g/m^2^. High stocking density inhibited growth performance and caused physiological response. These insights offer guidance for the selection of an appropriate stocking density for *P. fulvidraco* in integrated rice–fish farming systems.

**Abstract:**

*Pelteobagrus fulvidraco* is a freshwater fish commonly raised in rice fields, yet the optimal stocking density for this species remains unknown. Therefore, this study aimed to investigate the appropriate stocking density of *P. fulvidraco* in integrated rice–fish farming systems. Three different stocking densities––low density (LD, 125 g/m^2^), middle density (MD, 187.5 g/m^2^), and high density (HD, 250 g/m^2^)––were set up to evaluate *P. fulvidraco*’s growth performance, stress indices, immune function, antioxidant status, and lipid metabolism after 90 days of farming. The results indicated that HD treatment had a detrimental effect on *P. fulvidraco*’s growth parameters. HD treatment led to an increase in cortisol (Cor) and lactate (La) levels, but a decrease in glucose (Glu) content in serum. After 90 days of farming, an immune response accompanied by the increase of complement 3 (C3), C4, and immunoglobulin M (IgM) was observed in the HD group. Meanwhile, HD treatment induced oxidative stress and altered antioxidative status evidenced by the levels of catalase (CAT), glutathione peroxidase (Gpx), glutathione (GSH), malondialdehyde (MDA), superoxide dismutase (SOD), and total antioxidant capacity (T-AOC) in serum or liver. Additionally, the lipid metabolism-related genes including lipoprotein lipase (*lpl*), peroxisome proliferators-activated receptor (*pparα*), carnitine palmitoyltransferase-1 (*cpt-1*), and sterol regulatory element binding protein-1 (*srebp-1*) were markedly downregulated in the HD and/or MD group after 90 days of farming. In conclusion, this study contributes to a better understanding of *P. fulvidraco*’s response to different stocking densities in integrated rice–fish farming systems. We suggest that the appropriate stocking density for *P. fulvidraco* in these farming systems should be below 250 g/m^2^, considering both fish growth and physiological responses.

## 1. Introduction

Aquaculture is an increasingly important source of protein and plays a vital role in global food security [1]. In 2021, China’s aquaculture yield reached 53.94 million tons, accounting for approximately 60% of the world’s yield [2]. Various aquaculture models are used in China, including pond farming, cage farming, recirculating aquaculture, and integrated rice–fish farming [3]. To ensure sustainable aquaculture, it is necessary to improve the utilization rate of water and land resources as well as to reduce the environmental pollution [4]. Integrated rice–fish farming is an efficient ecological model that is in line with the development of green ecological agriculture, with significant economic, ecological, and social benefits [5,6]. This farming model greatly reduces environmental impacts and improves resource utilization [7,8]. In the system, fish feces improve soil fertility, which reduces the need for fertilizers [9], while plankton in rice fields provides a rich diet for fish [10]. Fish farming also helps to control weeds, pests, and diseases in rice fields [11]. Integrated rice–fish farming has been successfully practiced with species such as common carp (*Cyprinus carpio*) [12], largemouth bass (*Micropterus salmoides*) [13], tilapia (*Oreochromis niloticus*) [14], crayfish (*Pmcambarus clarkii*) [15], and Chinese mitten crab (*Eriocheir sinensis*) [16]. It has become a modern agricultural production model that utilizes an ecological cycle and achieves high quality and efficiency [10].

Stocking density is a pivotal factor affecting growth performance, physiological function, behavior, and welfare in aquatic animals [17]. In aquaculture practice, in order to increase productivity and profits, aquafarms often resort to increasing stocking density. However, an excessively high stocking density may result in negative consequences and decrease economic benefits from aquaculture [18]. A previous study has shown that high stocking densities (10.92 g/m^2^) can lead to decreased survival rate, weight, and specific growth rate of weight in *O. niloticus* (18.2 ± 0.17 g, 4 months) in rice–fish farming systems [19]. A similar result was found for GIFT (genetically improved farmed tilapia) tilapia (*Oreochromis niloticus*) (24.18 g/m^2^, 12.09 g, 92 d) culturing in rice–fish systems [20]. Additionally, the growth and survival rates of carp (*Cyprinus carpio*) (74.96 g/m^2^, 100 g, 90 d) [21] and *M. salmoides* (120 g/m^3^, 40.63 ± 0.13 g, 90 d) [22] were negatively impacted under high stocking density culturing in rice–fish systems. Under high stocking density, increased competition for food and living space can also cause significant physiological stress [23], which requires more energy to maintain physiological balance and resist stress reaction [24]. Reports suggest that high stocking density causes oxidative stress that leads to malondialdehyde (MDA) content increased in aquatic animals like crayfish (*Cherax quadricarinatus*) (107.19 g/m^2^, 14.29 ± 1.05 g, 90 d) culturing in aquariums [25]. Moreover, it has been observed that high stocking density can reduce the immunity of aquatic species, leading to higher rates of disease infection [26,27]. Therefore, selection of the appropriate stocking density for aquatic animals is crucial in aquaculture operations.

The yellow catfish (*P. fulvidraco*) is one of the most important cultivated freshwater fish species in China, with a production of 565,477 and 587,822 tons in 2020 [28] and 2021 [29], respectively. Previous studies on *P. fulvidraco* were limited to cage culture [30], recirculating aquaculture systems [31], and ponds [32], which revealed varying stocking densities for optimal growth. For example, the appropriate stocking density is 216–270 g/m^3^ (15–20 g/individual) in ponds [33,34], 2–3.5 kg/m^2^ (20–50 g/individual) in cages [35,36], and 1 kg/m^3^ (25 g/individual) in recirculating aquaculture systems [37]. Recently, researchers have compared growth performance and muscular quality of *P. fulvidraco* in ponds and paddy fields [38]. However, the suitable stocking density of *P. fulvidraco* in the integrated rice–fish farming system is still unclear. Therefore, this study aims to explore the suitable stocking density for *P. fulvidraco* in the integrated rice–fish farming system via comparing the differences of growth performance, physiological function, antioxidant status, and lipid metabolism. The results will provide crucial reference for determining stocking density of *P. fulvidraco* in rice field cultivation.

## 2. Materials and Methods

### 2.1. Fish and Experimental Design

The *P. fulvidraco* used in the study was provided by the farm of Freshwater Fisheries Research Center (Jingjiang, China). All fish were in good health and had an average body weight of 145.3 ± 5.19 g. The experiment was conducted at the same farm. According to the previous studies on the farming practice of *Pelteobagrus fulvidraco* in the integrated rice-fish farming systems in China [39,40], three stocking densities of *P. fulvidraco* were set: low density (LD, 125 g/m^2^, 344 individuals), middle density (MD, 187.5 g/m^2^, 516 individuals) and high density (HD, 250 g/m^2^, 688 individuals), with three repetitions per group. In the experiment, each replicate group consists of two zones, including a rice field (340 m^2^) and a canal refuge (1.2 m in depth, 60 m^2^). In the paddy, rice (Nangeng 5055) was planted at the start of July 2021, and harvested at the start of November. *P. fulvidraco* was stocked at the end of July 2021 and harvested at the end of October 2021. We added suitable water into the system to keep the stability of water volume when it decreased due to evaporation. In canal refuge, the aerator was installed to maintain the water oxygen level. During the experiment, commercial feed (HAID Group Co., Ltd., Guangzhou, China) was fed once daily, with the amount approximately 1% of their weight. The main nutrient composition of the diet included 42.0% crude protein, 5.0% crude fat, 3.0% crude fiber, 16.0% crude ash, and 0.80% total phosphorus. During the trial, the water’s dissolved oxygen was 4.4–5.0 mg/L, the pH was 7.41–8.05, and the temperature was 22.3–33.5 °C.

### 2.2. Samples Collection

For water quality analysis, we collected a water sample every 30 days according to the five-point sampling method [41]. Each repetition collected five samples. To evaluate the growth performance of *P. fulvidraco*, 90 fish in each group were caught randomly every 30 days to measure weight and body length.

At the end of the experiment, 36 fish were randomly caught from each group and anesthetized with 50 mg/L MS-222 (Fujian Shengyuan Aquatic Products Co., Ltd., Ningde, China). The blood was collected via caudal vein and centrifuged at 3600 r/min for 10 min to obtain serum. After blood sampling, the liver tissue was collected for biochemical analysis and gene expression. The blood and liver tissues from 4 fish were mixed to form one sample. All sample were stored at −80 °C. The liver was taken into a 1.5 mL centrifuge tube (0.1 g of the liver was added to 0.9 mL 0.86% physiological saline) to grind. Then, the homogenate was centrifuged at 3000 g for 10 min. Finally, the supernatant was collected to assay antioxidant parameters. The study was authorized by the Freshwater Fisheries Research Center (FFRC) of the Chinese Academy of Fishery Sciences (CAFS).

### 2.3. Water Quality Analysis

Total phosphorus (TP) was detected according to the ammonium molybdate spectrophotometric method [42]. Total nitrogen (TN) was tested according to the alkaline potassium persulfate digestion-UV spectrophotometry [43]. The Nessler’s reagent colorimetric method was selected for the determination of ammonium (NH_4_^+^-N) [44]. The spectrophotometric method was used to measure nitrite (NO_2_^−^-N) [45]. Nitrate (NO_3_^−^-N) was determined via the spectrophotometric method with phenol disulfonic acid [46].

### 2.4. Growth Parameters Analysis

The growth performance was assessed by analyzing weight gain (WG, %), specific growth rate of weight (SGR, %), condition factor (CF, g/cm^3^), and survival rate (SR, %). The specific calculation formulas are as follows:

WG = 100 × (W_2_ − W_1_)/W_1_

SGR = 100 × (lnW_2_ − lnW_1_)/T

CF = 100 × W_2_/L_2_^3^

SR = 100 × final number/initial number

where, W_1_ and W_2_ are initial body weight (g) and final body weight (g), L_2_ is the final body length (cm), and T is the total trial days (d).

### 2.5. Serum Physiological Parameters Analysis

Serum physiological parameters, including total cholesterol (TC), triglyceride (TG), glucose (Glu), low-density lipoprotein cholesterol (LDL-c), and high-density lipoprotein cholesterol (HDL-c), were determined using automatic biochemical analyzer (BS-400, Mindray Biomedical, Shenzhen, China). The lactate (La) was measured by a commercial reagent kit (Nanjing Jiancheng Bioengineering Institute, Nanjing, China). Cortisol (Cor), heat shock protein 70 (HSP70), complement 3 (C3), complement 4 (C4), lysozyme (LZM), and immunoglobulin M (IgM) were determined using enzyme linked immunosorbent assay (Shanghai Enzyme-linked Biotechnology Co., Ltd., Shanghai, China). The determination of all parameters was conducted according to the method described by the manufacturers.

### 2.6. Antioxidant Status Analysis

In order to assess the impact of stocking density on the antioxidant status of *P. fulvidraco*, a range of parameters were measured using commercially available kits. Specifically, the levels of catalase (CAT), glutathione peroxidase (Gpx), glutathione (GSH), malondialdehyde (MDA), superoxide dismutase (SOD), total antioxidant capacity (T-AOC), and total protein were measured in both the serum and liver of the fish. Commercial kits were used for each assay, including Beyotime Biotechnology (Shanghai, China) for CAT (References: S0051) and MDA (References: S0051) [47,48], Grace Biotechnology Co., Ltd. (Suzhou, China) for Gpx (References: S0051) [49], and Nanjing Jiancheng Bioengineering Institute (Nanjing, China) for GSH (References: S0051), SOD (References: A001-3), T-AOC (References: A015-3), and total protein (References: A045-4) [13,50]. The determination of all parameters was conducted according to the method described by the manufacturers.

### 2.7. Gene Expression Analysis

According to the manufacturer’s instructions, Trizol reagent (Takara Biomedical Technology Co., Ltd., Beijing, China) was used to extract total RNA from liver. The RNA purity and integrity were evaluated by calculating the ratio of OD_260_/OD_280_ (1.7–2.1) and detecting gel electrophoresis. The total RNA was then reverse transcribed into cDNA using a commercial kit (Takara, RR047A). Firstly, 1 μg total RNA was mixed into 2 μL 5× gDNA Eraser Buffer, 1 μL gDNA Eraser and appropriate amount RNase Free ddH_2_O to remove genome DNA under 42 °C 2 min (total volume 10 μL). Then, the mixture was mixed into 1 μL PrimeScript RT Enzyme Mix I, 1 μL RT Primer Mix, 4 μL 5× PrimeScript Buffer, and 4 μL RNase Free dH_2_O to perform reverse transcription using PCR system (ABI, Foster, CA, USA). The reverse transcription procedure consisted of the following parts: 37 °C for 15 min, and 85 °C for 5 s.

To detect the expression of the target gene, a commercial kit (Takara, RR820A) was used for quantitative real-time PCR (qPCR) amplification on a CFX96 Real-Time PCR Detection System (Bio-Rad, Hercules, CA, USA). The CFX Maestro software was used for data collection, analysis, and visualization. The PCR reaction solution consisted of 2 μL cDNA, 12.5 μL TB Green *Premix Ex Taq* II, 1 μL PCR forward and reverse primers (10 μM), and 8.5 μL RNase Free ddH_2_O. The PCR thermal cycling conditions were as follows: an initial denaturation step of 95 °C for 30 s, followed by 40 cycles of denaturation (95 °C, 5 s), and annealing and extension (60 °C, 30 s), followed by melt curve (65–95 °C) at the end of the last cycle. The specific primers used in qPCR are shown in Table 1.

The mathematical model Bestkeeper [51] was used to compare the stability of reference genes (*β-actin*, *gapdh* and *18s rRNA*) [52,53,54]. According to Ct values (Table S2), we calculated the correlation coefficient (r), standard deviation (SD), and coefficient of variation (CV) of the reference genes under different groups. A reference gene with high r value and low SD and CV values is considered to be more stable. Moreover, when SD > 1, it indicates that the reference gene is unstable. The analysis results showed that *β-actin* is more stable compared with *18s rRNA* and *gapdh* under different densities (Table S3). Therefore, the *β-actin* was chosen as a reference gene, and the relative expression levels of the target gene were calculated by 2^−ΔΔCt^ method [55].

**Table 1:** Gene-specific qPCR primers.

Gene	F: Primer Sequence (5′-3′)	R: Primer Sequence (5′-3′)
*β-actin* [52]	GTACCACCATGTACCCTGGC	GTGCCTTTCATTCAGCCACC
*lpl* [56]	GACCAGAGAGATGATGCCGT	TAGCTTAGCTGGCTCTTGCTG
*pparα* [57]	CGAGGATGGGATGCTGGTG	CGTCTGGGTGGTTCGTCTGC
*cpt-1* [57]	ATTTGAAGAAGCACCCAGAGTATGT	CCCTTTTATGGACGGAGACAGA
*srebp-1* [57]	CTGGGTCATCGCTTCTTTGTG	TCCTTCGTTGGAGCTTTTGTCT
*gapdh* [53]	CACTGCCACCCAGAAGACA	AGGGACACGGAAAGCCAT
*18s rRNA* [54]	CCTGAGAAACGGCTACCACATCC	AGCAACTTTAATATACGCTATTGGAG

### 2.8. Statistical Analysis

The data were analyzed using SPSS 25.0, and the results were expressed as mean ± standard deviation (SD). The Shapiro–Wilk test and Levene test were used to evaluate the normal distribution and variance homogeneity of experimental data, respectively. The difference among different stocking densities was analyzed by one-way ANOVA with LSD post hoc test. It was considered statistically significant if the *p*-value < 0.05.

## 3. Results

### 3.1. Change of Water Quality Parameters

**Figure 1:**
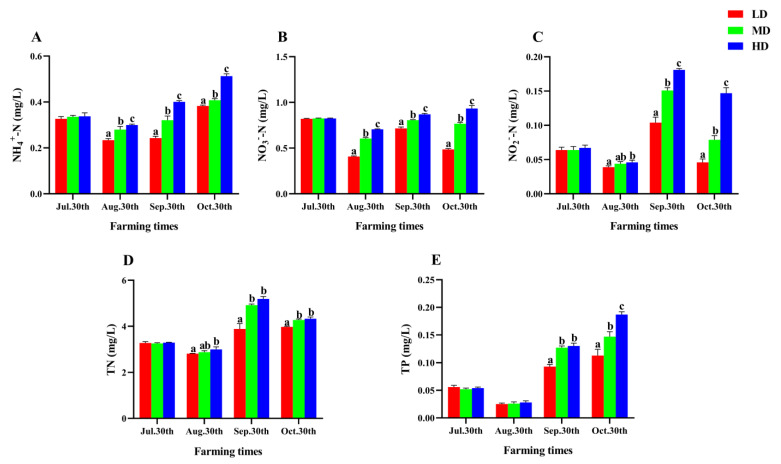
Water quality parameters of *P. fulvidraco* reared at different densities in an integrated rice–fish farming system. NH_4_^+^-N (**A**), NO_3_^−^N (**B**), NO_2_^−^N (**C**), TN (**D**), and TP (**E**). Values are presented as means ± SD (*n* = 3). Different letters as superscripts indicate significant differences among the different groups (*p* < 0.05). LD, low stocking density; MD, medium stocking density; HD, high stocking density.

The change of water quality parameters in each group was shown in Figure 1. Throughout the experiment, the levels of NH_4_^+^-N, NO_3_^−^-N, NO_2_^−^-N, TN, and TP exhibited varying changes from 30 July to 30 October. The contents of NO_2_^−^-N and TN reached the maximum on 30 September, while the contents of NH_4_^+^-N and TP gradually increased after August 30 and reached the maximum on October 30. In addition, all nutrient contents in the HD group were significantly higher than those in the LD group after 90 days of farming (*p* < 0.05, Figure 1).

### 3.2. Change of Growth Performance

**Figure 2:**
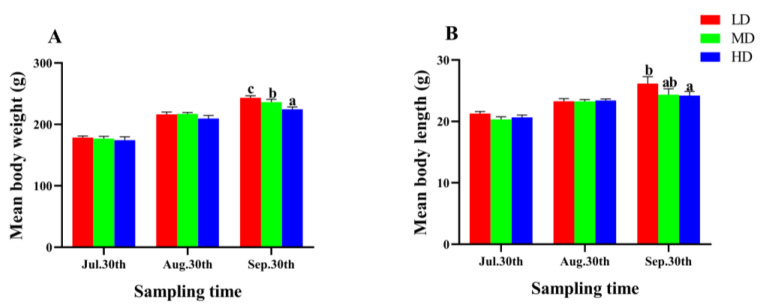
Mean body weight (**A**) and mean body length (**B**) of *P. fulvidraco* reared at different densities in an integrated rice–fish farming system. Values are presented as means ± SD (*n* = 3). Different letters as superscripts indicate significant differences among the different groups (*p* < 0.05). LD, low stocking density; MD, medium stocking density; HD, high stocking density.

After 90 days of fish farming, the WG and SGR of *P. fulvidraco* were significantly lower in the HD group compared to the MD and LD groups (*p* < 0.05, Table 2). However, the CF and SR showed no significant difference among different groups (*p* > 0.05, Table 2). As the farming time increased, the mean body length and weight of *P. fulvidraco* in each group presented an upward trend (Figure 2), and the two parameters in the LD group were significantly higher than that in the HD group at the end of the trial (*p* < 0.05, Figure 2).

### 3.3. Change of Stress Indices

**Figure 3:**
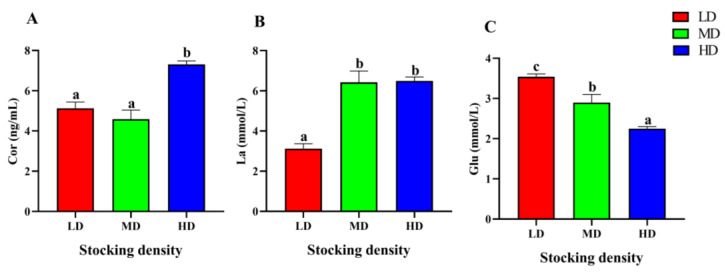
Stress parameters of in the serum *P. fulvidraco* reared at different densities in an integrated rice–fish farming system. Cor (**A**), La (**B**), and Glu (**C**). Values are presented as means ± SD (*n* = 3). Different letters as superscripts indicate significant differences among the different groups (*p* < 0.05). LD, low stocking density; MD, medium stocking density; HD, high stocking density.

At the end of the trial, the serum stress parameters of *P. fulvidraco* under different stocking densities had significant differences (Figure 3). The content of Cor in the HD group was markedly higher than that in the LD and MD groups after 90 days of farming (*p* < 0.05). Compared with the LD group, the content of La in the MD and HD increased significantly (*p* < 0.05). In addition, with the increase of stocking density, the level of Glu in serum decreased significantly (*p* < 0.05).

### 3.4. Change of Lipid Metabolism

**Figure 4:**
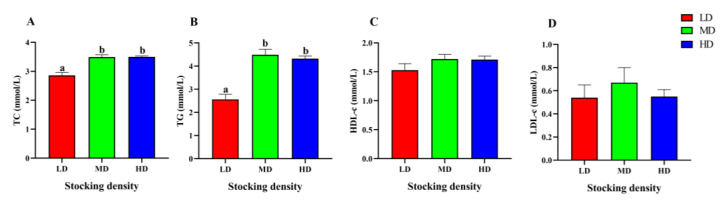
Lipid metabolism parameters in the serum of *P. fulvidraco* reared at different densities in an integrated rice–fish farming system. TC (**A**), TG (**B**), HDL-c (**C**), and LDL-c (**D**). Values are presented as means ± SD (*n* = 3). Different letters as superscripts indicate significant differences among the different groups (*p* < 0.05). LD, low stocking density; MD, medium stocking density; HD, high stocking density.

The changes of lipid metabolism parameters in the serum of *P. fulvidraco* under different densities were shown in Figure 4. Compared with the LD group, the content of TC and TG in the MD and HD groups increased significantly (*p* < 0.05). However, different stocking densities had no significant effect on the levels of TC, HDL-c, and LDL-c in serum (*p* > 0.05).

### 3.5. Change of Immune Function

**Figure 5:**
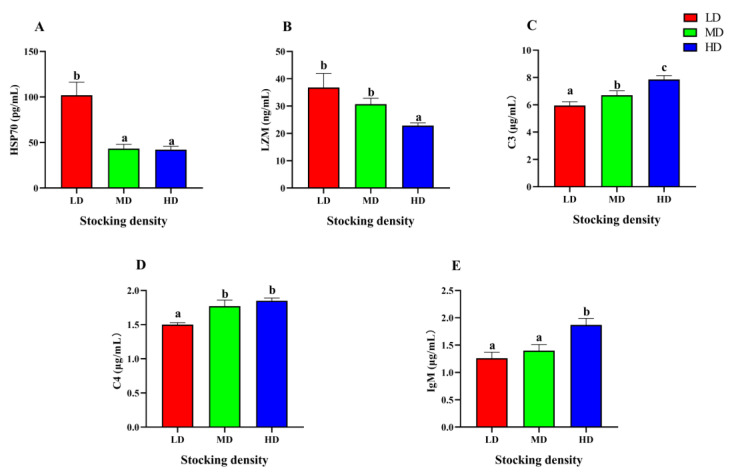
Immune parameters in the serum of *P. fulvidraco* reared at different densities in an integrated rice–fish farming system. HSP70 (**A**), LZM (**B**), C3 (**C**), C4 (**D**), and IgM (**E**). Values are presented as means ± SD (*n* = 3). Different letters as superscripts indicate significant differences among the different groups (*p* < 0.05). LD, low stocking density; MD, medium stocking density; HD, high stocking density.

After 90 days farming, significant differences were observed in the serum immune parameters of *P. fulvidraco* under different densities (Figure 5). The content of HSP70 in the MD and HD groups was significantly lower than that in the LD group, while the content of LZM in the HD group was significantly lower than that in the LD and MD groups (*p* < 0.05). As the stocking density increased, there was a corresponding increase in the levels of C3, C4, and IgM. Specifically, the HD group exhibited significantly higher levels of C3, C4, and IgM when compared to the LD group (*p* < 0.05).

### 3.6. Change of Antioxidant Status

**Figure 6:**
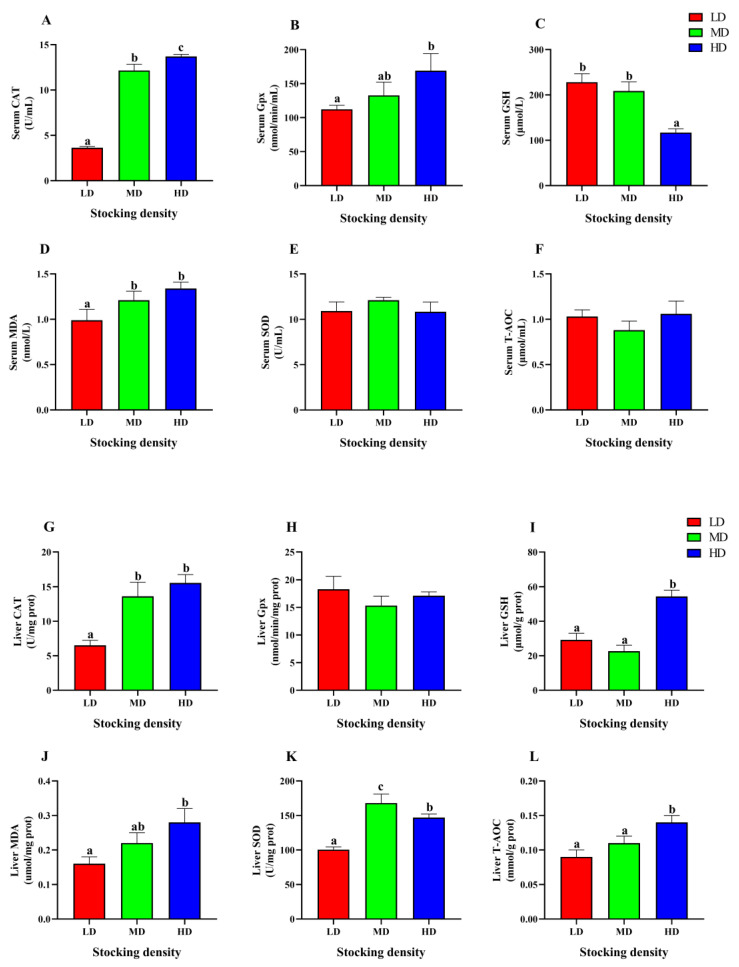
Antioxidative parameters in serum (**A**–**F**) and liver (**G**–**L**) of *P. fulvidraco* reared at different densities in an integrated rice-fish farming system. Serum CAT (**A**), Serum Gpx (**B**), Serum GSH (**C**), Serum MDA (**D**), Serum SOD (**E**), Serum T-AOC (**F**), Liver CAT (**G**), Liver Gpx (**H**), Liver GSH (**I**), Liver MDA (**J**), Liver SOD (**K**) and Liver T-AOC (**L**). Values are presented as means ± SD (*n* = 3). Different letters as superscripts indicate significant differences among the different groups (*p* < 0.05). LD, low stocking density; MD, medium stocking density; HD, high stocking density.

In the serum, the HD group showed significantly higher activities of CAT and Gpx, as well as MDA content, compared to the LD group (*p* < 0.05, Figure 6A,B,D). However, the GSH content was markedly decreased in the HD group compared to the LD group (*p* < 0.05, Figure 6C). In addition, there was no significant difference observed in SOD and T-AOC levels among the different groups (*p* > 0.05, Figure 6E,F).

In the liver, the levels of CAT, SOD, T-AOC, GSH, and MDA in the HD group were significantly higher than that in the LD group (*p* < 0.05, Figure 6G,I–L), but the activity of Gpx did not show any significant difference among the different groups (*p* > 0.05, Figure 6H).

### 3.7. Change of Liver Gene Expression

**Figure 7:**
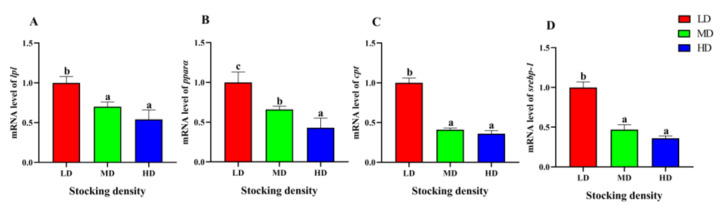
Gene expression in the liver of *P. fulvidraco* reared at different densities in an integrated rice–fish farming system. *lpl* (**A**), *pparα* (**B**), *cpt* (**C**), and *srebp-1* (**D**). Values are presented as means ± SD (*n* = 3). Different letters as superscripts indicate significant differences among the different groups (*p* < 0.05). LD, low stocking density; MD, medium stocking density; HD, high stocking density.

The relative gene expression of *lpl* and *pparα* in the HD and MD groups were significantly lower than that in the LD group in the liver (*p* < 0.05, Figure 7). Similarly, the relative gene expression of *cpt* and *srebp-1* in the MD and HD groups was significantly lower than that in the LD group (*p* < 0.05, Figure 7).

## 4. Discussion

### 4.1. Effect of Stocking Density on Water Quality

Water quality is a crucial factor in the success of aquaculture. Poor water quality can lead to increased stress in aquatic animals, resulting in reduced survival and growth rates [58,59]. The relationship between stocking density and water quality parameters in various aquaculture systems has been widely reported. Studies have found that stocking density has a significant impact on water quality, and that the levels of water quality parameters gradually increase with increasing stocking density in pond farming, which could be responsible for the growth inhibition of *C. idella* (2570 ± 102.96 g, HD: 15 kg/m^3^, 120 d) and *M. amblycephala* (95.47 ± 6.30 g, HD: 10 kg/m^3^, 120 d) in ponds [60]. However, Liu et al. (2022) have shown that the stocking density does not impact water quality in the integrated rice–fish farming system [61], which is because rice can absorb nutrients and improve water quality [62]. In our study, we found that the water quality parameters, including TP, TN, NH_4_^+^-N, NO_3_^−^-N, and NO_2_^−^-N, were significantly higher in the HD and MD groups than the LD group after 90 days of farming. We attribute this to the increased dissolution of excrement and feed in the water under high stocking density.

The absorption capacity of rice to N and P is different at different rice growth stages [10]. During the tillering stage, rice displays a strong ability to assimilate N and P, effectively reducing the N and P load in water and soil [63,64,65]. Our study revealed that rice demands a substantial amount of N and P during the tillering stage to support its growth, resulting in a decrease in N and P content (30th August). However, during non-irrigation periods, the absorption and purification capacity of rice attenuates, leading to an upsurge in N and P concentration in water. Notably, paddy fields represent a confined ecosystem, with its small water volume highly susceptible to temperature and precipitation fluctuations [66,67]. Consequently, nutrient concentrations displayed a fluctuating trend during the experiment.

### 4.2. Effect of Stocking Density on Growth Performance

It was reported that the stocking density is one of the critical factors of fish growth performance [68]. A previous study showed that SGR, FCR, and protein efficiency ratio (PER) of spotted sea bass (*Labeo bata*) (203.27 ± 26.55 g) were negatively impacted by high stocking densities (618 g/m^3^, 14 d), which led to decreased growth performance [69]. Debnath et al. (2016) found that Indian butter catfish (*Ompok bimaculatus*) (0.83 ± 0.02 g), reared at lower density (3.32 g/m^2^, 8 months) demonstrated significant improvements in body length, weight, and survival rates compared to those reared at higher density (4.98 g/m^2^, 8 months) [70]. Similarly, in this study, the body weight, body length, SGR, and WG in the HD group were significantly lower than that in the LD group after 90 days of farming, indicating that high stocking density inhibited the growth of *P. fulvidraco*. This effect is likely due to increased competition for food and living space caused by increased social interaction among fish. It was reported that increased social interaction caused significant differences in individual size [71,72,73]. Wickins et al. (2010) reported that the growth performance of the juvenile European eel (*Anguilla anguilla*) (0.26 g) in an aquarium was significantly improved after it was removed from high stocking density (247 g/m^3^, 86 d) and isolated [74]. Although higher stocking density is often seen as a way to improve the utilization of water and land resources, it may not necessarily lead to increased production [75]. Research on juvenile turbot (*Scophthalmus maximus*) raised in PVC tanks (8.62 ± 0.06 g), for example, suggests that high stocking densities (1.8 kg/m^3^, 45 d) can prolong growth periods and reduce yield by decreasing SGR [76]. Given the potential economic risks, there is a need for further study and attention to optimal stocking densities in aquaculture activities [77].

### 4.3. Effect of Stocking Density on Stress Response

High stocking density is a common stressor that disrupts physiological functions [78]. Cor is a well-known indicator of stress response [79,80] and is produced through hypothalamus-pituitary-interrenal tissues in fish under stress [81]. It has been reported that a large amount of energy was generated to resist external stress, when Cor acts on carbohydrate metabolism, proteolysis, and lipid oxidation [80,82]. However, prolonged elevation of Cor levels can cause serious cell damage [83]. La is also a crucial biomarker for stress response in aquatic animals [84]. For the juvenile flounder (*Paralichthys orbignyanus*) raised in tanks, the serum Cor was significantly increased at high stocking density (7.8 kg/m^3^, 15 d) [85]. Similar results were observed in gilthead sea bream (*Sparus aurata*) (89.5 ± 21.1 g, 70–90 kg/m^3^, 45 d) [86] and Channel catfish (*Ictalurus punctatus*) (40.6 ± 2.23 g, 12.18 kg/m^3^, 60 d) [87] in the high-density group. Our study on *P. fulvidraco* also revealed increased Cor level in the HD group and increased La level in the MD and HD groups after 90 days of farming, highlighting the chronic stress caused by inappropriate stocking density.

Glucose has been widely recognized as a common indicator for assessing the level of stress [88]. It has been observed that the elevation of cortisol content facilitates metabolic stimulation, increases gluconeogenesis, and promotes the accumulation of glucose [89]. Previous findings have indicated that acute crowding stress (70 kg/m^3^, 3 h) can result in hyperglycemia in *C. carpio* (70.69 ± 8.11 g) raised in cages [90]. However, in this study, serum Glu content of *P. fulvidraco* decreased significantly with the increase of stocking density. One possible explanation for this phenomenon is that high stocking density intensifies the competition for food and living space amongst individuals, thereby leading to strenuous physical activity and increased glucose consumption.

### 4.4. Effect of Stocking Density on Lipid Metabolism

Lipid metabolism is a physiological process that is often altered during periods of stress, as the body expends extra energy. Blood lipid levels, such as TC, TG, HDL-c, and LDL-c are commonly used as parameters for evaluating lipid metabolism [91]. In *M. salmoides* (9.71 ± 3.75 g) raised in ponds, high stocking density (1.94 kg/m^3^, 180 d) led to a significant increase in the TG level, but the TC level did not exhibit any significant differences among different stocking densities, suggesting that high stocking density may accelerate lipid metabolism [92]. In a previous study on *I. punctatus* (40.6 ± 2.1 g, 12.18 kg/m^3^, 60 d) raised in tanks, the TC, TG, and HDL-c levels in the high-density group were significantly higher than those in the low-density group, while the LDL-c level in the medium density group were significantly higher than those in the low-density group, likely due to increased energy demands at high stocking densities [89]. Our study also revealed a marked increase in TC and TG levels in both the HD and MD groups, indicating that lipid metabolism may be enhanced to produce more TC and TG for energy consumption under high stocking density conditions.

We further found that the expression of four pivotal genes associated with lipid metabolism including lipoprotein lipase (*lpl*), peroxisome proliferators-activated receptor (*pparα*), carnitine palmitoyltransferase-1 (*cpt-1*), and sterol regulatory element binding protein-1 (*srebp-1*) in the liver of *P. fulvidraco* were significantly altered under different stocking densities. LPL is a crucial enzyme involved in the process of lipid deposition and metabolism [93,94], while PPARα plays a major role in stimulating lipid catabolism and maintaining cholesterol homeostasis [95,96]. Cpt-1 is the rate-limiting enzyme affecting fatty acid β-oxidation [97], and SREBP-1, in conjunction with PPARα, participates in regulating the synthesis of unsaturated fatty acids [98,99]. Previous studies have shown that under high stocking density, the mRNA level of *lpl* was reduced in amur sturgeon (*Acipenser schrenckii*) (225.5 ± 32.3 g, 11.0 kg/m^3^, 70 d) and *C. idella* (98.48 ± 6.00 g, 14.9 kg/m^3^, 70 d), indicating that lipid synthesis was inhibited [100,101]. The mRNA level of *pparα* in the liver of *A. schrenckii* increased significantly with the increase of stocking density, indicating that the lipid utilization ability was enhanced under high stocking density, resulting in a decrease in lipid accumulation [100]. Contrary result was found in the research on *M. salmoides*, indicating a disturbance in lipid metabolism under high density stress [13]. Several studies showed that the expression of *cpt-1* and *srebp-1* in fish can easily be influenced by stress. For instance, the mRNA levels of *cpt-1* was inhibited significantly in *M. salmoides* (10.87 ± 0.17 g, 60 d) exposed to hypoxia stress [102] and in tilapia exposed to tetracycline for a long time [103]. Similarly, *srebp-1* expression was down-regulated in the liver of *S. maximus* (197 ± 3.68 g, 48 h) subjected to low salinity stress [104]. In this study, the HD and MD treatments induced a down-regulation of *lpl*, *pparα, cpt-1,* and/or *srebp-1*, indicating that stress caused by high stocking condition may disrupt lipid metabolism in the liver.

### 4.5. Effect of Stocking Density on Immune Performance

The immune system of fish is a vital defense mechanism against disease. Immune response is a common phenomenon that helps fish cope with adverse stimuli. LZM, C3, C4, IgM, and HSP70 are well studied immune parameters in fish [105]. LZM is involved in gram-positive cell wall lysis [106], while C3 is considered an intersection between nonspecific and adaptive immunity, activating the complement through the classical, alternative, and lectin pathways [107]. C4 plays a role in the neutralization of viruses via classical complement activation [108,109], whereas IgM is the first immunoglobulin found in fish and is important in acquired immunity [110]. HSP70 is an inducible heat shock protein that mitigates cellular damage by repairing and degrading denatured proteins [111,112]. As a chaperone molecule, HSP70 plays important roles in metabolism and cell survival [113]. Numerous studies have confirmed that stocking density affects immune parameters. Specifically, the level of LZM significantly decreased with increasing stocking density in aquatic animals, such as turbot (*Scophthalmus maximus*) [114] and Chinese sturgeon (*Acipenser sinensis*) [115]. The effect of stocking density on C3 and C4 content varies between species. For instance, C3 content was observed to increase significantly under high stocking density of *I. punctatus* [87], while it decreased significantly with increasing stocking density of Russian sturgeon (*Acipenser gueldenstacdti*) [116]. A recent study showed that the expression of *hsp70* in the liver of *I. punctatus* was markedly reduced while the level of IgM increased significantly under high stocking density [87]. Our study found that the levels of C3 and C4 in the HD and MD groups, and IgM in the HD group were significantly higher than that in the LD group after 90 days of farming, indicating that the high stocking density induced *P. fulvidraco* to respond to external stimulation by activating immune system. Meanwhile, the levels of LZM in the HD group and HSP70 in the MD and HD groups were markedly lower compared with the LD group. We speculated that the LZM was consumed and HSP70 synthesis was inhibited under an unsuitable stocking density. In addition, the decrease of HSP70 may affect cell proliferation [117].

### 4.6. Effect of Stocking Density on Antioxidative Status

Numerous studies have demonstrated that multiple stressors can cause an intracellular redox imbalance, which induces oxidative stress in fish [118]. Long term or severe oxidative stress may disturb the antioxidant defense system and lead to cell damage [119]. High stocking density has been shown to decrease levels of antioxidant parameters, such as SOD, CAT, Gpx, GSH, and T-AOC, thereby damaging the antioxidant defense system in several fish species, including *M. salmoides* (4.50 ± 0.23 g, 0.6 kg/m^3^, 60 d) [120] raised in ponds and *M. amblycephala* (25.76 ± 2.25 g, 3.09 kg/m^3^, 6 weeks) raised in cages [121]. Conversely, some studies suggest that high stocking density can lead to an increase in antioxidant levels in golden pompano (*Trachinotus ovatus*) (9.75 ± 0.11 g, 2.93 kg/m^3^, 70 d) raised in cages [122], jaw characin (*Salminus brasiliensis*) (5.67 g, 1.70 kg/m^3^, 80 d) raised in ponds [123], and large yellow croaker (*Larimichthys crocea*) (89.06 ± 11.70 g, 34.2 kg/m^3^, 72 h) raised in aquariums [124]. These findings indicated that more antioxidant enzymes were produced to eliminate oxygen free radicals under high density stress [125]. In this study, the HD and MD groups showed a marked increase in the levels of T-AOC, GSH, CAT, and/or SOD in the liver after 90 days of farming, which indicated that the antioxidant defense system was activated to maintain ROS balance under a high stocking density.

MDA is the final product of lipid peroxidation, and it can combine with free amino acids to cause crosslink polymerization of macromolecules, including proteins and nucleic acids, leading to cellular injury [126,127]. Under normal physiological status, the content of MDA in fish is low [128]. However, exposure to stressors like high stocking density can significantly increase MDA content [120,121]. Refaey et al. have demonstrated that the MDA content of *I. punctatus* reared in high density increased markedly compared to low density conditions [87]. Moreover, *S. maximus* (185.4 g) raised in recirculating aquaculture system also displayed a marked increase in MDA formation at high-density group (19.1 kg/m^2^, 120 d) [129]. Similarly, in this study, chronic stress induced by high stocking density caused increased lipid peroxidation, leading to a significant rise in MDA content in the serum and liver of fish after 90 days of farming.

## 5. Conclusions

Our findings indicated that the *P. fulvidraco* is a suitable species for the integrated rice–fish farming system. However, we observed that growth parameters were reduced when the stocking density of *P. fulvidraco* exceeded 250 g/m^2^ in the integrated rice–fish farming system. In addition, although the MD group did not show differences in growth compared to LD, some evidence of suffering stress was found, which indicated that this density seems to be inappropriate for longer rearing periods (higher than 90 days). Our data also revealed that MD and/or HD treatments resulted in increased physiological stress, which was evidenced by alterations in lipid metabolism, immune function, and antioxidant status. Consequently, *P. fulvidraco* required additional energy to maintain a balance in its physiological state. These results suggested that the growth inhibition of *P. fulvidraco* in high density stocking conditions may be attributed to a degradation of water quality and changes in physiological status due to increased interspecific interaction. These insights offer guidance for selection of an appropriate stocking density for *P. fulvidraco* in integrated rice–fish farming systems.

## Figures and Tables

**Table 2:** Growth parameters of *P. fulvidraco* at different stocking densities after 90 days of farming.

Groups	WG (%)	SGR (%/d)	CF (g/cm^3^)	SR (%)
LD	67.46 ± 2.34 ^b^	0.57 ± 0.01 ^b^	1.37 ± 0.16	84.01 ± 3.30
MD	62.60 ± 3.19 ^b^	0.54 ± 0.02 ^b^	1.64 ± 0.16	83.59 ± 2.13
HD	54.63 ± 2.53 ^a^	0.48 ± 0.02 ^a^	1.59 ± 0.14	81.97 ± 1.29

Note: The same column of data with different letters indicate that the difference is significant (*p* < 0.05). Values are presented as means ± SD (*n* = 3). LD, low stocking density; MD, medium stocking density; HD, high stocking density.

## Data Availability

Not applicable.

## Supplementary Materials

The following supporting information can be downloaded at: https://www.mdpi.com/article/10.3390/ani13111721/s1, Table S1: Gene-specific qPCR primers. Table S2: Ct values of the three reference genes. Table S3. Bestkeeper analysis of reference genes from liver in *Pelteobagrus fulvidraco.* Refs. [51,52,53,54] are cited in the Supplementary Materials.
