# Supplementary material for: Effects of Stocking Density on the Growth Performance, Physiological Parameters, Antioxidant Status and Lipid Metabolism of Pelteobagrus fulvidraco in the Integrated Rice-Fish Farming System
Source: Animals (Basel). 2023 May 23;13(11):1721. doi: 10.3390/ani13111721 (PMC10251942; doi:10.3390/ani13111721)
Supplement: Supplementary file 1 [file animals-13-01721-s001.zip › supplementary material S2.zip!/supplementary material S2.pdf]

Effects of stocking density on the growth performance, physiological parameters, antioxidant status and lipid metabolism of *Pelteobagrus fulvidraco* in the integrated rice-fish farming system

Weixu Diao [1,†], Rui Jia [1,2,3,†], Yiran Hou [1,2], Yin Dong[1], Bing Li [1,2,*] and Jian Zhu [1,2,*]

[1] Wuxi Fisheries College, Nanjing Agricultural University, Wuxi 214081, China; 2021113006@stu.njau.edu.cn (W.D.); jiar@ffrc.cn (R.J.); houyr@ffrc.cn (Y.H.)

[2] Key Laboratory of Integrated Rice-Fish Farming Ecology, Ministry of Agriculture and Rural Affairs, Freshwater Fisheries Research Center, Chinese Academy of Fishery Sciences, Wuxi 214081, China

[3] International Joint Research Laboratory for Fish Immunopharmacology, Freshwater Fisheries Research Center, Chinese Academy of Fishery Sciences, Wuxi 214081, China

* Correspondence: lib@ffrc.cn (B.L.); zhuj@ffrc.cn (J.Z.); Tel.: +86-85550414 (B.L.); +86-85550535 (J.Z.).

† These authors contributed equally to this work.

The stability of reference genes analysis

In the study, we selected three common reference genes: *β-actin*, *18s rRNA* and *gapdh* (Table S1) to analyze the stability using Bestkeeper software (Ingrid A Aursnes, Anne L Rishovd, Hans E Karlsen, & Gjøen, 2011).

According to Ct values (Table S2), we calculated the correlation coefficient (r), standard deviation (SD), and coefficient of variation (CV) of the reference genes under different group. Reference gene with high r value and low SD and CV values is considered to be more stable. Moreover, when SD > 1, it indicates that the reference gene is unstable.

The analysis results showed that *β-actin* is more stable compared with *18s rRNA* and *gapdh* under different densities (Table S3).

Table S1. Gene-specific qPCR primers.

Gene	F: primer sequence (5'-3')	R: primer sequence (5'-3')
β-actin (Guo et al., 2021)	GTACCACCATGTACCCTGGC	GTGCCTTTCATTCAGCCACC
gapdh (Song et al., 2015)	CACTGCCACCCAGAAGACA	AGGGACACGGAAAGCCAT
18s rRNA (Chen et al., 2015)	CCTGAGAAACGGCTACCACATCC	AGCAACTTTAATATACGCTATTG GAG

Table S2. Ct values of the three reference genes:

	β-actin	*18s rRNA*	*gapdh*
1	22.67	8.57	33.57

2	22.68	8.62	33.25
3	23.02	9.00	34.58
4	22.76	8.56	32.76
5	23.02	8.46	35.37
6	23.06	8.72	34.55
7	22.82	8.54	33.54
8	22.95	8.44	34.28
9	22.65	8.41	32.35
10	23.13	9.01	34.33
11	23.02	8.67	35.21
12	22.74	8.32	32.96
13	22.46	8.36	33.50
14	22.47	8.33	33.46
15	22.65	8.53	32.74
16	23.23	9.15	34.42
17	23.16	9.06	34.85
18	22.65	8.49	34.02
19	23.04	8.76	34.05
20	22.76	8.56	33.21
21	22.74	8.65	33.56
22	22.65	8.34	34.41
23	22.61	8.23	33.49
24	22.65	8.21	33.45
25	22.58	8.50	32.75
26	22.66	8.70	32.71
27	22.63	8.41	32.83

Note: LD:1-9, MD:10-18, HD:19-27.

Table S3. Bestkeeper analysis of reference genes from liver in *Pelteobagrus fulvidraco*

	β-actin	***18s rRNA***	***gapdh***
coeff. of corr. [r]	0.923	0.874	0.811
p-value	0.001	0.001	0.001
SD	0.19	0.19	0.69
CV	0.81	2.21	2.03

Note: Pair wise correlation analysis showing Pearson correlation coefficient (r), the value of probability (*p*), standard deviation (SD), and coefficient of variation (CV). Pair of genes with r > 0.80 in bold.

Reference

Chen, Q. L., Luo, Z., Shi, X., Wu, K., Zhuo, M. Q., Song, Y. F., & Hu, W. (2015). Dietary methimazole-induced hypothyroidism reduces hepatic lipid deposition by down-regulating lipogenesis and up-regulating lipolysis in *Pelteobagrus fulvidraco*. *Gen Comp Endocrinol, 217-218*, 28-36. doi:10.1016/j.ygcen.2015.05.006

Guo, J., Pu, Y., Zhong, L., Wang, K., Duan, X., & Chen, D. (2021). Lead impaired immune function and tissue integrity in yellow catfish (*Peltobargus fulvidraco*) by mediating oxidative stress, inflammatory response and apoptosis. *Ecotoxicol Environ Saf, 226*, 112857.

doi:10.1016/j.ecoenv.2021.112857

Ingrid A Aursnes, Anne L Rishovd, Hans E Karlsen, & Gjøen, T. (2011). Validation of reference genes for quantitative RT-qPCR studies of gene expression in Atlantic cod (*Gadus morhua l.*) during temperature stres. *BMC Research Notes, 4*, 104.

Song, Y. F., Luo, Z., Huang, C., Chen, Q. L., Pan, Y. X., & Xu, Y. H. (2015). Endoplasmic Reticulum Stress-Related Genes in Yellow Catfish *Pelteobagrus fulvidraco*: Molecular Characterization, Tissue Expression, and Expression Responses to Dietary Copper Deficiency and Excess. *G3 (Bethesda), 5*(10), 2091-2104. doi:10.1534/g3.115.019950
